# Supplementary material for: Content-rich biological network constructed by mining PubMed abstracts
Source: BMC Bioinformatics. 2004 Oct 8;5:147. doi: 10.1186/1471-2105-5-147 (PMC528731; doi:10.1186/1471-2105-5-147)
Supplement: Additional File 2 — The original results of the above study (non-essential files are deleted to keep the file size under the limit set by BMC bioinformatics). [file 1471-2105-5-147-S2.bz2 › chilibotAdditionalFile2/dip30/357ID9062193E4999/html/IL1R1_IL1B.html]

 


 **IL1R1** and **IL1B** 
  
Found 453 abstracts in PubMed, retrieved 30.  
 

 What does Google say? 
 PDF only 
| .edu only 

---

**Interactive relationship** (e.g. stimulation, inhibition, etc)

**Neutral relationship**- The observation that IL 1 type I receptor  [ **IL1R1** ]  was expressed in highest density in the hypothalamus was of significance in identifying a role for IL 1 beta  [ **IL1B** ]  in neuroendocrine modulation.  Ref: 11898392 Vitam Horm, 2002

**Non-interactive relationship** (e.g. studied together, co-existance, homology, etc.)

- DESIGN AND METHODS The associations of a variable number tandem repeat 86 bp polymorphism in intron 2 of interleukin 1 receptor  [ **IL1R1** ]  antagonist IL1 RA and of the 511 C T polymorphism of IL 1b  [ **IL1B** ]  with the risk of CAD were studied.  Ref: 12551827 Haematologica, 2003
- METHODS Interleukin 1 beta  [ **IL1B** ]  IL 1 beta  [ **IL1B** ]  and interleukin 1 receptor  [ **IL1R1** ]  antagonist IL 1Ra produced by cultured peripheral blood mononuclear cells PBMC after exposure to cuprammonium Cup membrane, polysulfone PS membranes or endotoxin were detected using enzyme linked immunoabsorbent assay.  Ref: 11775226 Chin Med J Engl , 2000
- OBJECTIVE Since ulcerative colitis and Crohn s disease, which are associated with ankylosing spondylitis AS, have been found to be variably associated with the IL 1B  [ **IL1B** ]  and the IL 1RN genes encoding interleukin 1beta IL 1beta and the interleukin 1 receptor  [ **IL1R1** ]  antagonist IL 1ra, we have investigated whether these polymorphisms in IL 1B  [ **IL1B** ]  and IL 1RN are also involved in AS.  Ref: 12468823 Rheumatology Oxford , 2002
- OBJECTIVE To study the effect of interleukin 1 beta  [ **IL1B** ]  and interleukin 8 in the pathogenesis of colitis and evaluate the therapeutic effect of interleukin 1 receptor  [ **IL1R1** ]  antagonist IL 1ra.  Ref: 11717998 Zhongguo Yi Xue Ke Xue Yuan Xue Bao, 1998
- OBJECTIVE To investigate the polymorphisms of interleukin 1B IL 1B  [ **IL1B** ]  promotor region 31 C T and interleukin 1 receptor  [ **IL1R1** ]  antagonist IL 1RN gene and the relationship between the genotype of IL 1 and IL 1RN and susceptibility to gastric cancer in Chinese population.  Ref: 12133467 Zhonghua Yi Xue Za Zhi, 2002
- OBJECTIVE We examined the association between preterm delivery and polymorphisms at position 3953 of the interleukin 1 beta  [ **IL1B** ]  gene  **IL1B**  3953 and in intron 2 of the interleukin 1 receptor  [ **IL1R1** ]  antagonist gene IL1RN.  Ref: 12114904 Am J Obstet Gynecol, 2002
- and 15 lung, polymorphisms in 8 genes that influence the inflammatory process, namely  **IL1B** ,  **IL1R1** , IL1RN, IL6, IL10, TNFA, TGFB1 and FCGRIIA, were examined.  Ref: 12095061 Am J Transplant, 2002
- Plasma and peripheral blood mononuclear cell PBMC production of interleukin 1 beta  [ **IL1B** ]  IL 1beta, tumor necrosis factor alpha TNF alpha, interleukin 6 IL 6, interleukin 1 receptor  [ **IL1R1** ]  antagonist IL 1ra and soluble TNF receptor type II sTNFrII were measured at baseline and, 24 and 168h following exercise.  Ref: 12161104 Cytokine, 2002
- The effect of interleukin 1 beta  [ **IL1B** ]  interleukin 8 in the pathogenesis of experimental colitis and evaluation of interleukin 1 receptor  [ **IL1R1** ]  antagonist therapy < ArticleTitle>.  Ref: 11717998 Zhongguo Yi Xue Ke Xue Yuan Xue Bao, 1998
- Expression of interleukin 1 beta  [ **IL1B** ]  and interleukin 1 receptor  [ **IL1R1** ]  antagonist in oxLDL treated human aortic smooth muscle cells and in the neointima of cholesterol fed endothelia denuded rabbits.  Ref: 12577317 J Cell Biochem, 2003
- The genetic relationship between IBD and the interleukin 1 receptor  [ **IL1R1** ]  antagonist and interleukin 1beta genes IL 1RN, and IL 1B  [ **IL1B** ] , respectively has been extensively studied.  Ref: 11686217 Scand J Gastroenterol, 2001
- CONCLUSION A relative inability to down regulate proinflammatory interleukin 1 beta  [ **IL1B** ]  activity by interleukin 1 receptor  [ **IL1R1** ]  antagonist may contribute to the pathophysiologic features of vulvar vestibulitis syndrome.  Ref: 11967493 Am J Obstet Gynecol, 2002
- Polymorphisms for interleukin 1 beta  [ **IL1B** ]  exon 5 and interleukin 1 receptor  [ **IL1R1** ]  antagonist in Taiwanese children with febrile convulsions.  Ref: 12038885 Arch Pediatr Adolesc Med, 2002
- The culture supernatants were then assayed for interleukin 1 beta  [ **IL1B** ]  and interleukin 1 receptor  [ **IL1R1** ]  antagonist by enzyme linked immunosorbent assay.  Ref: 11967493 Am J Obstet Gynecol, 2002
- Plasma and peripheral blood mononuclear cells PBMC were obtained from 55 healthy human volunteers, whose genotypes for four polymorphisms of IL1 family genes  **IL1B** ,  **IL1R1**  and IL1RN were determined by PCR.  Ref: 12355453 Eur J Immunol, 2002
- The ratio of interleukin 1 receptor  [ **IL1R1** ]  antagonist to interleukin 1 beta  [ **IL1B** ]  was higher in control subjects than in women with vulvar vestibulitis syndrome in response to both heat shock protein P =.0002 and lipopolysaccharide P =.01.  Ref: 11967493 Am J Obstet Gynecol, 2002
